# An active and targeted survey reveals asymptomatic malaria infections among high-risk populations in Mondulkiri, Cambodia

**DOI:** 10.1186/s12936-023-04630-2

**Published:** 2023-06-23

**Authors:** Dyna Doum, David J. Mclver, John Hustedt, Jeffrey Hii, Siv Sovannaroth, Dysoley Lek, Jason H. Richardson, Allison Tatarsky, Neil F. Lobo

**Affiliations:** 1 Health Forefront Organization, Phnom Penh, Cambodia; 2 Malaria Elimination Initiative, University of California, San Francisco, CA USA; 3 National Center for Parasitology, Entomology and Malaria Control, Phnom Penh, Cambodia; 4 Innovative Vector Control Consortium, Liverpool, UK; 5 Eck Institute for Global Health, University of Notre Dame, Notre Dame, USA

**Keywords:** Malaria, Asymptomatic, Blood spots, Prevalence, PCR, Plasmodium

## Abstract

**Background:**

Malaria is a mosquito-borne disease that is one of the most serious public health issues globally and a leading cause of mortality in many developing countries worldwide. Knowing the prevalence of both symptomatic and asymptomatic malaria on a subnational scale allows for the estimation of the burden of parasitaemia present in the transmission system, enabling targeting and tailoring of resources towards greater impact and better use of available capacity. This study aimed to determine the PCR-based point prevalence of malaria infection, by parasite species, among three high-risk populations in Mondulkiri province, Cambodia: forest rangers, forest dwellers, and forest goers.

**Methods:**

A cross-sectional survey was performed during the transmission season in November and December 2021. Blood samples collected on filter paper from participants (n = 1301) from all target groups were screened for *Plasmodium* spp using PCR.

**Results:**

Malaria prevalence among all study participants was 6.7% for any *Plasmodium* species. Malaria prevalence in the forest ranger group was 8.1%, was 6.8% in forest goers, and 6.4% in forest dwellers; all infections were asymptomatic. *Plasmodium vivax* was detected in all participant groups, while the few *Plasmodium falciparum* infections were found in goers and dwellers. 81% of all infections were due to *P. vivax*, 9% were due to *P. falciparum*, 3% due to *Plasmodium cynomolgi*, and the rest (7%) remained undefined. Gender was associated with malaria infection prevalence, with male participants having higher odds of malaria infection than female participants (OR = 1.69, 95% CI 1.08–2.64). Passively collected malaria incidence data from the Cambodian government were also investigated. Health facility-reported malaria cases, based on rapid diagnostic tests, for the period Jan-Dec 2021 were 521 *Plasmodium vivax* (0.89% prevalence)*,* 34 *P. falciparum* (0.06%) and four *P. falciparum* + mixed (0.01%)—a total of 559 cases (0.95%) for all of Mondulkiri.

**Conclusion:**

This reservoir of asymptomatic parasitaemia may be perpetuating low levels of transmission, and thus, new strategies are required to realize the goal of eliminating malaria in Cambodia by 2025.

**Supplementary Information:**

The online version contains supplementary material available at 10.1186/s12936-023-04630-2.

## Background

The Greater Mekong Subregion (GMS) in Southeast Asia has experienced dramatic declines in malaria transmission, with 65,297 cases reported in 2021, representing a 16% decrease from 2020 [1]. Cambodia in particular has seen a dramatic drop in malaria cases in the past decade; in just 1 year, from 2020 to 2021, confirmed malaria cases have decreased by 54% [1]. This decrease is partly due to targeted treatment and management activities outlined in the National Strategic Plan for Elimination of Malaria 2011–2025 [2, 3]. Accordingly, the National Center for Entomology, Parasitology and Malaria Control (CNM) has developed an elimination strategy and plan towards eliminating malaria by 2025 [3, 4]. The Global Technical Strategy for Malaria 2016–2030 elevates surveillance as a core intervention in all malaria-endemic settings [5]. Cambodia has two surveillance systems to report malaria cases: the Health Management Information System and the Malaria Information System (MIS). Cambodia had partitioned the malaria-endemic areas into four zones based on their yearly parasite index and malarial multi-drug resistance status. With the recent reduction of malaria, these have been collapsed into non-endemic and elimination areas. Both types depend on quality diagnosis and treatment at health facilities, village malaria workers, and vector control by promoting malaria prevention education and distributing long-lasting insecticide nets. The non-endemic zones, where no local transmission had been recently reported, uses government-run healthcare facilities’ essential services for diagnosis and treatment [4]. The zone with the most malaria incidence in Cambodia and across GMS is predominantly found in the North-Eastern regions such as Ratanakiri and Mondulkiri [6]. These settings are characterized by a high proportion of ethnic minorities, with agricultural or forest-related activities being primary income sources. High malaria risk is seen in forested areas and in sub-populations engaging in forest-related activities exposed to the *Anopheles* mosquito bites [7, 8].

Knowing the point prevalence of both symptomatic and asymptomatic infections on a subnational scale allows for the estimation of the parasite reservoir in the transmission system [9], enabling targeting and tailoring of resources towards greater impact and better use of available capacity. In Cambodia, malaria incidence is based on symptoms in conjunction with a positive malaria test—typically rapid diagnostic tests (RDTs) or microscopy. Asymptomatic malaria parasite carriers will therefore not present for malaria diagnosis, resulting in this portion of the reservoir not being detected and reported, even though they may serve as a significant reservoir of parasites [10, 11]. In addition, passive case detection misses infections where *Plasmodium* carriage is below the threshold of detection of the diagnostic method used [12, 13]. *Plasmodium* detection by PCR is much more sensitive compared to RDT or microscopy, particularly in low density or mixed infection cases, and it is valuable for the accurate collection of malaria epidemiological data [14, 15]. As countries approach elimination, there is greater need for accurate characterization of malaria parasites in both symptomatic and asymptomatic individuals [5]. The objective of this study was to determine the PCR-based point prevalence of malaria infection by parasite species in each of three target populations (forest rangers, forest dwellers, and forest goers) at high risk of malaria infection in Mondulkiri, Cambodia.

## Methods

### Study area and population

This study was conducted in Mondulkiri Province, Cambodia. Mondulkiri was the location for this study given its higher burden status relative to other provinces in Cambodia, and because Mondulkiri was the site for other related research activities under a broader vector control research program in collaboration with CNM. Villages were chosen based on ongoing local malaria passive case reporting (ongoing vector-based transmission) and consultations with local health authorities, with the 21 villages with the highest reported number of cases included in the study, with the addition of forest ranger stations which are associated with rangers who work in high-risk areas (the total number of ranger stations in the province is not publicized). Where active malaria transmission remains in Cambodia, the foci are largely related to forested areas, and forest exposure has been documented elsewhere as a risk factor for malaria in the country [8, 16]. Therefore, individuals at high risk of malaria infection in Mondulkiri province that were targeted for blood sample collection within the villages included:

- 1) Forest rangers and forest patrol teams, who spend at least 21 days/month in forested areas,
- 2) Forest dwellers, who live and work inside forests or within 1 km of forest edge, and.
- 3) Forest goers, who live more than 1 km from forest edge, but typically visit forested areas at least 1 day per week.

These locations and populations were specifically chosen for this study as a preliminary investigation into current levels of malaria in advance of a then-planned randomized controlled trial. The trial was intended to be run in the same locations and with the same at-risk populations as were selected in the present study. While the villages selected were chosen based on the number of malaria cases reported through the government tracking system, the province, operating district, and the particular at-risk populations were selected to conform with the planned trial.

Three sample size estimates were determined for each of the three target populations (Forest Rangers, Forest Dwellers, and Forest Goers) based on precision estimates of malaria infection, with a coefficient of variation of 0.5, a significance of 5%, and 80% power. All sample size estimates were powered to provide a baseline estimate of the prevalence of malaria by PCR for each population using the Hayes and Bennett (1999) approach. Based on sample size calculations for the three target groups combined, a total samples size of 1450 was determined.

### Cross-sectional survey

This study used a cross-sectional design targeting clusters with high rates of malaria, as reported to the district health offices and the national Malaria Information Service. The primary clusters for rangers were ranger stations and villages for forest goers and forest dwellers. Villages and ranger stations were randomly selected for inclusion in the study. Within selected ranger stations, all rangers who were willing to participate in the study were enrolled. In selected villages, household were selected using a simple random sample, and all eligible individuals living in that household were invited to participate. Inclusion criteria for study participants comprised of being in one of the target groups, weight of > 20 kg (included to reflect the inclusion criteria for a then-planned randomized controlled trial), ability to provide informed consent, and speaking Khmer or Bunong [17]. Study staff (Health Forefront Organization, a non-government organization) visited both households and ranger stations to screen for eligibility, obtain informed consent, administer questionnaires, and perform finger prick blood collection. The survey captured individual and household-level demographic information, use of vector control tools, history of malaria infection, and treatment-seeking behaviour. Informed consent was obtained from all participants, including parental consent for any individual younger than 18 years. If the participant was symptomatic (fever > 98°F) during the survey, a malaria RDT (SD Bioline Malaria Ag P.F/Pv) was also administered. If positive for malaria infection by RDT, the participant was to be referred to the nearest Village Malaria Worker, Mobile Malaria Worker, or health facility for treatment.

### Sample processing and Plasmodium detection by PCR

Blood samples were collected (~ 50 ul blood via finger prick), for a total of four dried blood spots (DBSs) per individual on filter paper (Whatman Filter Paper #3), from each participant at a single time point. All blood samples were screened for *Plasmodium* spp infection by the COX-III direct PCR method [18] using AccuStart II PCR ToughMix (Quantabio, 0.6–2 parasites per microliter limit of detection) at the University of Notre Dame, USA.

### Data analysis

The outcome measure was the malaria infection prevalence (by parasite species) per target population and was determined using the number of positive individuals divided by the total sample size. Univariable logistic regression analyses were used to identify potential risk factors for malaria infection against data collected via the questionnaire. All factors were included in the univariable analysis and variables that were related to malaria infection prevalence with a p-value of < 0.25 in the univariable analyses were subsequently combined in a multivariable model. A p-value of < 0.05 was considered statistically significant, and the corresponding odds ratios with 95% confidence interval were also estimated. STATA version 14.0 was used for data analysis.

### Health facility and village malaria worker data

Passive malaria incidence data is generated by village malaria workers (VMWs). VMWs are trained to diagnose malaria using RDTs and treat uncomplicated malaria cases [19]. Symptomatic villagers visit VMWs for RDT screening and malaria treatment if positive. VMWs record all cases that are tested, confirmed, and treated at the village level with data recorded on paper-based forms that are collated monthly from respective Operational Districts (ODs), electronically entered into the Malaria Information System (MIS), and sent to CNM, which appends the comprehensive, country-wide database [20]. Malaria reporting data from January to December 2021 was collected directly from MIS for the entire province of Mondulkiri. In addition, photographs of VMW-completed paper forms were taken from all villages included in the cross-sectional survey. The data from these forms was then digitized into Excel format and used for descriptive analysis of the quality and completeness of the recorded data, which is important for quantifying and identifying important risk factors or high-risk groups.

### Ethical considerations

This study was approved by the National Ethics Committee for Health Research of the Ministry of Health of Cambodia (ref. no. 241 NECHR) and the University of California, San Francisco (ref. no 21-34947). The purpose of the study was explained to all study participants and written informed consent was obtained. Participants understood that they were free to remove themselves from the study at any time without repercussion. All the data and samples were de-identified and coded towards analysis following IRB guidelines.

## Results

### Characteristics of the study population

**Fig. 1:**
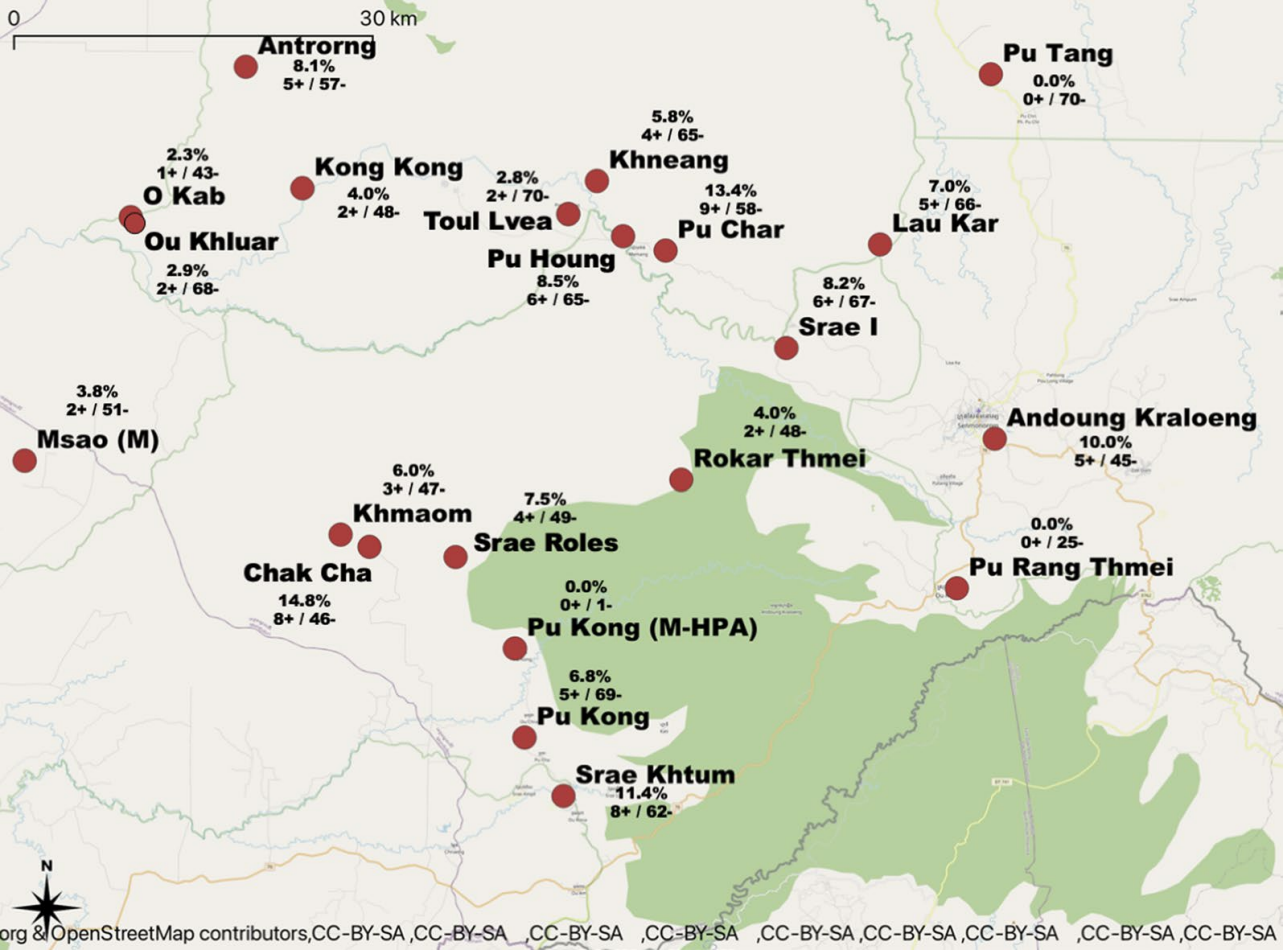
Proportion of PCR positive malaria samples in each study location (location of ranger stations are not permitted to be mapped)

A total of 1301 participants were recruited into the study, of which 770 (59.2%) were from the forest goer group, 469 (36.1%) from the forest dweller group, and 62 (4.7%) from the forest ranger group. Individuals were recruited from 21 different villages (Fig. 1), and rangers were from 15 different ranger stations (due to privacy concerns, locations of ranger stations are not mapped). Due to constraints in both time and funding, the estimated required sample size of 1450 was not met. Participant ages ranged from 4 to 99 years with a mean age of 31.5 years (SD 15.7). Proportion of participant gender was approximately equal (50.1% males, 49.9% females). Approximately half of the participants belonged to the indigenous minority Bunong group (52.7%), 44.4% were Khmer, and 3.0% were of other ethnic groups. In addition, 546 (42.0%) of the participants had never been diagnosed with malaria before, while 215 (16.5%) had received one malaria diagnosis in their life, and 540 (41.5%) people had been diagnosed with malaria more than once. Of the participants who had been diagnosed with malaria at least once, they reported that their most recent diagnosis was within: previous week (4, 0.3%), previous month (13, 1.0%), previous three months (20, 1.5%), previous six months (8, 0.6%), previous year (76, 5.8%), or was more than 1 year ago (622, 47.8%). Of the three groups of participants, forest rangers reported the highest proportion of participants who had been diagnosed with malaria at least once (80.7%). Descriptive demographic details of the study population are shown in Table 1.

**Table 1:** Study group demographics

Variables	N = 1301		
	Forest goer (%)	Forest dweller (%)	Forest ranger (%)
No. of individuals	770 (59.19)	469 (36.05)	62 (4.77)
Age group (year)			
4–10	51 (6.62)	27 (5.76)	0 (0.00)
11–20	194 (25.19)	107 (22.81)	0 (0.00)
21–30	163 (21.17)	130 (27.72)	22 (35.48)
31–40	119 (15.45)	89 (18.98)	26 (41.94)
41–50	122 (15.84)	50 (10.66)	12 (19.35)
≥ 51	121 (15.71)	66 (14.07)	2 (3.23)
Gender			
Male	358 (46.49)	232 (49.47)	61 (98.39)
Female	412 (53.51)	237 (50.53)	1 (1.61)
Ethnic group			
Khmer	361 (46.88)	168 (35.82)	48 (77.42)
Bunong	382 (49.61)	295 (62.90)	8 (12.90)
Other	27 (3.51)	6 (1.28)	6 (9.68)
Diagnosed with malaria*			
Never	362 (47.01)	172 (36.67)	12 (19.35)
1 time	124 (16.10)	82 (17.48)	9 (14.52)
2–5 times	213 (27.66)	157 (33.48)	21 (33.87)
6–10 times	42 (5.45)	33 (7.04)	6 (9.68)
≥ 11 times	29 (3.77)	25 (5.33)	14 (22.58)
Last time diagnosed with malaria*			
Never	245 (31.82)	138 (29.42)	11 (17.74)
Last week	2 (0.26)	2 (0.43)	0 (0.00)
Last month	9 (1.17)	4 (0.85)	0 (0.00)
Last 3 months	11 (1.43)	9 (1.92)	0 (0.00)
Last 6 months	5 (0.65)	3 (0.64)	0 (0.00)
Last year	52 (6.75)	23 (4.90)	1 (1.61)
More than 1 year	316 (41.04)	256 (54.58)	50 (80.65)
Missing data	130 (16.88)	34 (7.25)	0 (0.00)
Sources of family income in past year			
Farmer	543 (47.34)	402 (58.09)	17 (21.52)
Forest collector/forager	308 (26.85)	191 (27.60)	3 (3.80)
Day laborer	121 (10.55)	15 (2.17)	0 (0.00)
Ranger	1 (0.09)	1 (0.14)	58 (73.42)
Other	174 (15.17)	83 (11.99)	1 (1.27)
Frequency of travel outside village/station			
Daily	460 (59.75)	311 (66.31)	33 (53.23)
A few times a week	172 (22.34)	87 (18.55)	24 (38.71)
Weekly	49 (6.36)	16 (3.41)	2 (3.23)
Twice a month	24 (3.12)	13 (2.77)	2 (3.23)
Once a month	24 (3.12)	15 (3.20)	1 (1.61)
Less than once a month	41 (5.32)	27 (5.76)	0 (0.00)
Primary purpose for travel			
Work in the forest	195 (25.32)	98 (20.90)	61 (98.39)
Work at the farm	349 (45.32)	301 (64.18)	0 (0.00)
Work (other location)	87 (11.30)	5 (1.07)	0 (0.00)
Visiting family/friends	35 (4.55)	15 (3.20)	1 (1.61)
Other	104 (13.51)	50 (10.66)	0 (0.00)
Average hours walked when leaving village/station			
< 1 h	382 (49.61)	254 (54.16)	0 (0.00)
1–2 h	247 (32.08)	97 (20.68)	0 (0.00)
2–6 h	90 (11.69)	52 (11.09)	16 (25.81)
> 6 h	51 (6.62)	66 (14.07)	46 (74.19)
Visited a health facility over the past month			
Yes	227 (29.48)	116 (24.73)	10 (16.13)
No	542 (70.39)	353 (75.27)	51 (82.26)
Don’t Know	1 (0.13)	0 (0.00)	9 (1.61)

^*^It is recognized that the number of responses for “Number of times diagnosed with malaria = Never” (12) and “Last time diagnosed with malaria = Never” (11) are not equal for Rangers. There was one ranger who reported never having been diagnosed with malaria, but also reported having been diagnosed with malaria in the past year. At the time of data collection, this inconsistency was not identified, and therefore the data is reported here as it was collected

Of particular note is that the Forest Rangers represent a fairly different population compared to the Forest Goers and Forest Dwellers in the reason for spending time in the forest. Rather than spending time in forests for foraging, logging, or farming, rangers have extended patrols in forested areas and monitoring of protected wildlife and habitats. While the rangers do represent a different demographic in many ways, these differences were considered for further analyses, particularly in relation to infection rate, as discussed below (Table 2).

**Table 2:** Malaria prevalence confirmed by PCR

Target group	PCR positivity (%)	*Plasmodium*			*Undefined species*
		*vivax*	*falciparum*	*cynomolgi*	
Forest goer	52 (6.75)	40 (5.19)	6 (0.78)	2 (0.26)	4 (0.52)
Forest dweller	30 (6.40)	25 (5.33)	2 (0.43)	1 (0.21)	2 (0.43)
Forest ranger	5 (8.06)	5 (8.06)	0 (0.00)	0 (0.00)	0 (0.00)
Total	87 (6.69)	70 (5.38)	8 (0.61)	3 (0.23)	6 (0.46)

### Prevalence of Plasmodium spp

Overall, 87/1301 (6.7%) of participants were positive for any *Plasmodium* species, confirmed by PCR (Table 2. The malaria prevalence among the forest ranger group (8.1%) was higher than the forest goer (6.8%) and forest dweller (6.4%) group, though not statistically significantly so. The malaria prevalence among males (8.29%) was significantly higher than among females (5.08%) (OR = 1.69, 95% CI 1.08–2.64, univariable analysis). *Plasmodium vivax* infections were predominant across all participant groups, while the few *P. falciparum* infections were found only in the forest goer (0.8%) and dweller groups (0.4%). Infections with a simian malaria species, *P. cynomolgi*, were also found in both the forest goer group (n = 2) and the dweller group (n = 1). Six *Plasmodium* species were not determined, with four from forest goers and two from the dweller group.

### Associations between malaria infection prevalence and potential risk factors

.In both the univariable and multivariable analyses, only gender was found to be associated with malaria infection prevalence, where female participants had lower odds of being malaria positive compared to male participants (univariable analysis: OR = 0.59, 95% CI 0.37–0.92 and Adjusted OR = 0.61, 95% CI 0.38–0.97) (Table 3). Exploratory analyses investigated the role of target group (Forest Goers, Forest Dwellers, Forest Rangers), age, travel frequency, vector control tool use, and all variables listed in Table 1 and Additional file 1: Appendix 1, and none showed significant associations with infection in either univariable or multivariable analyses. A multivariable analysis with predictor variables of gender, age, target group, previous diagnosis with malaria, and frequency of travel outside village is also presented in Table 3 to explore potential confounding effects on the relationship between gender and malaria infection (Table 3).

**Table 3:** Risk factors association with malaria infection prevalence (n = 1301)

Variables	PCR positivity (%)	Univariate	P-value	Multivariate	
		OR (95% CI)		Adjusted OR (95% CI)	P-Value
Participant group					
Forest goer	52 (6.75)	Ref.			
Forest dweller	30 (6.40)	0.94 (0.59–1.50)	0.807	0.95 (0.59–1.54)	0.863
Forest ranger	5 (8.06)	1.21 (0.46–3.15)	0.695	1.09 (0.34–3.46)	0.874
Age group (year)					
4–10	4 (5.13)	Ref.			
11–20	23 (7.64)	1.53 (0.51–4.56)	0.445	1.59 (0.52–4.78)	0.409-
21–30	19 (6.03)	1.18 (0.39–3.59)	0.761	1.12 (0.36–3.48)	0.837
31–40	13 (5.56)	1.08 (0.34–3.44)	0.886	0.98 (0.30–3.19)	0.977
41–50	12 (6.52)	1.29 (0.40–4.13)	0.667	1.20 (0.36–3.93)	0.761
≥ 51	16 (8.47)	1.71 (0.55–5.29)	0.351	1.58 (0.50–4.97)	0.433
Gender					
Male	54 (8.29)	Ref.			
Female	33 (5.08)	0.59 (0.37–0.92)	**0.021**	0.61 (0.38–0.97)	**0.038**
Ethnic group					
Khmer	35 (6.07)	Ref.		Ref.	
Bunong	52 (7.59)	1.27 (0.81–1.98)	0.288		
Other	0 (0.00)	1	–		
Diagnosed with malaria					
Never	32 (5.86)	Ref.		Ref.	
1 time	12 (5.58)	0.94 (0.47–1.87)	0.882	0.97 (0.48–1.95)	0.952
2–5 times	30 (7.67)	1.33 (0.79–2.23)	0.273	1.27 (0.74–2.16)	0.373
6–10 times	6 (7.41)	1.28 (0.51–3.17)	0.587	1.21 (0.48–3.05)	0.685
≥ 11 times	7 (10.29)	1.84 (0.78–4.35)	0.163	1.71 (0.69–4.25)	0.244
Travel outside village					
Daily	55 (6.84)	Ref.			
A few times a week	17 (6.01)	0.87 (0.49–1.52)	0.628	–	–
Weekly	5 (7.46)	1.09 (0.42–2.84)	0.847	–	–
Twice a month	3 (7.69)	1.13 (0.33–3.80)	0.838	–	–
Once a month	3 (7.50)	1.10 (0.32–3.69)	0.872	–	–
Less than once a month	4 (5.88)	0.85 (0.29–2.42)	0.763	–	–
Primary purpose for travel					
Work in the forest	22 (6.21)	Ref.			
Work at the farm	47 (7.23)	1.17 (0.69–1.98)	0.544	–	–
Work (other location)	6 (6.52)	1.05 (0.41–2.67)	0.914	–	–
Visiting family/friends	4 (7.84)	1.28 (0.42–3.89)	0.658	–	–
Other	8 (5.19)	0.82 (0.35–1.90)	0.654	–	–
Travel outside village (average hours walked)					
Less than 1 h	39 (6.13)	Ref.			
1–2 h	27 (7.85)	1.30 (0.78–2.16)	0.307	1.29 (0.77–2.17)	0.327
2–6 h	10 (6.33)	1.03 (0.50–2.11)	0.927	0.96 (0.46–2.03)	0.932
More than 6 h	11 (6.75)	1.10 (0.55–2.21)	0.772	1.00 (0.45–2.21)	0.988
Visited a health facility over the past month					
Yes	25 (7.08)	Ref.			
No	62 (6.55)	0.92 (0.56–1.48)	0.735	–	–
Don’t know	0 (0.00)	1			

### Health facility based data

Passively reported malaria data was collected from the government’s Malaria Information System (MIS), which collects malaria case data from all villages in Mondulkiri. These health facilities reported the highest malaria caseloads in Mondulkiri in 2019–2020, which was the reason they were selected for this study. As part of the reporting, a total of 58,584 symptomatic (temperature > 98°F) patients from Mondulkiri were screened for malaria in 2021, of which 559 (0.95%) were RDT positive. Thirty-four (0.06%) were *P. falciparum* infections while 521 (0.89%) were *P. vivax*, and four (0.01%) were mixed infections (Table 4).

**Table 4:** Malaria cases, based on RDTs, from health facility records from Mondulkiri in 2021

	Health facility RDT positive			
Total tests	Pf	Pv	Mix	Total
58,584	34 (0.06%)	521 (0.89%)	4 (0.01%)	559 (0.95%)

## Discussion

In this study, a malaria prevalence survey was conducted among high-risk populations, including forest-goers, forest dwellers, and forest rangers living in, traveling to, and/or working in and around the forests who are exposed to *Anopheles* bites, to explore the reservoir of asymptomatic parasitaemia as the country accelerates towards malaria elimination. Data on asymptomatic parasitaemia among specific populations can support programmatic decision-making on appropriate malaria control measures to clear those reservoirs, including more targeted prevention interventions, such as bite prevention tools and chemoprevention approaches.

All malaria PCR-positive individuals in the survey were asymptomatic (temperature < 98°F). The overall PCR-based prevalence of malaria in this sample was 6.7%—of which, 80.5% of the cases were *P. vivax*, 9.2% were *P. falciparum*, 3.5% were *P. cynomolgi*, and 6.9% were of an undefined *Plasmodium* species due to inadequate genetic material or sample degradation—for sequencing. By comparison, a previous study in Mondulkiri conducted from 2017–2018 among the high risk population living outside the forest, at the forest fringe, and inside the forest, found an 8.3% point prevalence (349 infections/4200 population) [8]. The most recent PCR-based prevalence study from neighbouring Ratanakiri province reported a prevalence of 4.9% (244/4999), from similar at-risk populations, in 2012 [19]. The significant association of gender with increased risk of infection in these specific targeted high-risk populations is seemingly context-specific and likely dependent on differential exposure. In this study, data demonstrated that males, regardless of target group or reported activities, were at higher risk of malaria infection than females, and similar findings have previously been reported [20–23]. Previous studies have shown that males are likely to be more exposed to local malaria vectors due to behaviours including farming and forest activities, working with the upper body uncovered, and staying outside late at night with no bed net protection [21, 24–27].

This study supports prior molecular data which demonstrated that *P. vivax* infections predominate over *P. falciparum* in Cambodia in high-incidence provinces [8, 28–31]. Higher proportions of asymptomatic *P. vivax* infections, which may be regularly observed as relapses (though this was not investigated in this study), can lead to higher immunity levels and, thus, overall lower parasite density [32, 33]. To address *P. vivax* reservoirs and relapses, radical cure with primaquine (currently the only WHO-approved drug that clears *P. vivax* hypnozoites) is necessary. Administration of primaquine has significant limitations, including serious haemolysis risks in individuals with glucose‐6‐phosphate‐dehydrogenase enzyme (G6PD) deficiency [34].

There are access and delivery challenges associated with both G6PD testing and primaquine administration, including availability, non-compliance with treatment regiments, and/or side effects, among others. Despite these challenges, countries across the GMS, including Cambodia, are actively working to scale up implementation of G6PD testing and primaquine use to address *P. vivax* reservoirs and accelerate progress toward malaria elimination.

Individuals with asymptomatic infections generally remain undiagnosed and untreated as they do not present to health facilities, and consequently may remain infective to mosquitoes over long periods [35]. Individuals with asymptomatic malaria infections are thus silent reservoirs and pose a challenge to elimination efforts [36]. Data in the Malaria Information System (MIS) [37], operated by the Cambodia National Centre for Parasitology, Entomology, and Malaria Control, is collected by passive identification of malaria cases reported by VMWs (which is provided to the Operational District, then to the Provincial Health Department, an on to MIS). For all of 2021, there were 559 positives from 58,584 tests (0.95%) based on RDTs administered to symptomatic patients who reported to VMWs or health facilities from January to December. In comparison, the cross-sectional prevalence survey described here, using sensitive molecular diagnostics, found a much higher relative number of malaria infections [87 positive PCRs from 1301 individuals, or 6.7%) at a single timepoint than passive surveillance over an entire year. Due to differing definitions of what constitutes a “village” between the cross-sectional study and the MIS database (in some cases sub-villages may be considered as part of one larger village or they may be considered smaller villages unto themselves), and the timeframes available for MIS datasets, a direct comparison of cases from the exact same villages, over the same time period, was not possible. This greater sensitivity found in the cross-sectional study is attributed to asymptomatic infections being missed by the health system, the lower diagnostic sensitivity with RDTs [38] compared to PCR, as well as the active targeting of a higher risk population (forest goers, forest dwellers and rangers). This active targeting of high-risk groups using sensitive diagnostics highlights the importance of targeted surveillance activities to guide tailored response to ultimately interrupt malaria transmission—especially where the targeting of the asymptomatic reservoir may be crucial in an elimination setting.

PCR analyses detected three simian *P. cynomolgi* human infections, similar to another report in Cambodia from 2018, which was the first to report asymptomatic infections with this malaria parasite in the country [39]. Human infection with *P. cynomolgi* is not new, but it is highly uncommon in Cambodia [40–44]. This parasite was first discovered in 1907 in blood specimens collected from a cynomolgus monkey. In 1962, Cambodian monkeys were found also to be infected with *P. cynomolgi* and it was concluded that *Anopheles balabacensis* (now *Anopheles dirus*) probably was the vector of both human and monkey malaria and that the risk of cross infection was considerable if monkey malarias infective to humans exist in the Pailin area [45].

The morphological features of *P. cynomolgi*, as observed by microscopy, are almost identical to that of *P. vivax*, and *P. cynomolgi* also presents hypnozoites which can initiate relapses [46]. The persistence of a monkey reservoir of malaria parasites may confound the malaria elimination goal if *P. cynomolgi* is included in the WHO definition of elimination as a human parasite. As human malaria becomes more controlled and prevalence falls, primate malaria parasites may comprise an increasing proportion of cases in areas where monkeys live close to people [39].

It is of interest to note that this study was carried out during the COVID-19 pandemic, during which time many of the villages in Mondulkiri were on self-imposed lock down, before, during, and/or after the research was complete. The study team worked closely with local health officials and village leaders and were granted access to villages that may otherwise have been off limits to outsiders. While a thorough analysis and report has not been completed by the Cambodian government, there is a possibility that the COVID-19 pandemic may have impacted how malaria cases were detected, and ultimately the number of reported cases, both globally and in Cambodia [46]. The majority of malaria cases in the Mondulkiri area are identified when an individual self-reports to a VMW or a health center when feeling unwell, with an RDT used to confirm or rule out malaria. With the impact of COVID-19 not being spared in these villages, and peoples’ awareness of disease transmitting in their midst heightened, one might expect that the number of individuals self-reporting may have been higher than usual, and thus, more malaria cases could have been identified than otherwise, even with co-infections. However, local reports indicate that there were lower levels of malaria during the pandemic, with several potential reasons put forward, including:

A) The main focus of health workers during this time was on COVID-19 screening, and not on malaria;
B) With lockdowns imposed, people who would normally be travelling to and spending significant amounts of time in the forest, where they were at higher risk of malaria infection, were no longer doing so;
C) VMWs were less active during this time, owing to the restrictions on movement and of supplies;
D) Due to heightened awareness of disease transmission, people were potentially less likely to visit others in the community, including VMWs. It is unknown how the COVID-19 pandemic may have influenced the main finding of this paper, that active and targeted surveillance identifies more malaria cases than the existing passive surveillance system. However, if it is indeed true that there were fewer malaria cases than if the COVID-19 pandemic had not occurred, then the findings of this study could potentially be underestimated.

These findings highlight challenges that are faced by many malaria elimination programmes, including asymptomatic parasite reservoirs, *P. vivax* predominating, and gaps in passive surveillance. Targeting high-risk populations with more sensitive diagnostics and prevention and treatment interventions could substantially reduce the infectious reservoir [47, 48].

## Conclusion

Compared with standard, passive malaria case detection using RDTs on symptomatic individuals, active and targeted malaria detection using more sensitive PCR testing confirms that there is more malaria circulating in specific high-risk groups than previously assumed. This asymptomatic and untreated reservoir may be perpetuating low levels of malaria transmission and could slow progress towards elimination. New strategies targeted to high-risk populations that combine drug-based approaches, including chemoprevention and *P. vivax* radical cure, with tailored bite prevention or vector control tools will help clear these reservoirs and support near-term elimination goals in Cambodia and across the GMS.

## Supplementary Information

**Additional file 1: Appendix 1.** Cross-sectional Survey.

## Data Availability

All data and materials are available upon request from the corresponding author.

## Abbreviations

CNM: National Center for Entomology, Parasitology and Malaria Control
DBS: Dried blood spot
DOT: Directly observed treatment
GMS: Greater Mekong Subregion
MIS: Malaria information system
OD: Operational district
RDT: Rapid diagnostic test
VMW: Village malaria worker
