# Supplementary material for: An active and targeted survey reveals asymptomatic malaria infections among high-risk populations in Mondulkiri, Cambodia
Source: Malar J. 2023 Jun 23;22:193. doi: 10.1186/s12936-023-04630-2 (PMC10290344; doi:10.1186/s12936-023-04630-2)
Supplement: Supplementary file 1 — Additional file 1: Appendix 1. Cross-sectional Survey. [file 12936_2023_4630_MOESM1_ESM.docx]

Appendix 1 – Cross-sectional Survey

| **Question/Field** | **Responses** |
| --- | --- |
| 1. Supervisor name |  |
| 2. Village name |  |
| 3. Household code |  |
| 4. Individual code |  |
| 4. Household head name |  |
| 5. Date |  |
| 6. Participant Group |  |
|  |  |
| Household Roster |  |
| A1. What is your age? | Integer |
| A2. What is your Gender | Male |
|  | Female |
| A3a. What ethnic group do you identify with? | Khmer |
|  | Cham |
|  | Phnong/Bunong |
|  | Vietnamese |
|  | Kroeng |
|  | Tumpoun |
|  | Charay |
|  | Prouv |
|  | Kachak |
|  | Kavet |
|  | Lao |
|  | Lun |
|  | Other |
| A3b. Describe Other | Text |
| A.4. Do you spend time in the forest? | Yes |
|  | No |
| A.5a. How many nights do you spend in the forest per week, on average, during the dry season? | Integer |
| A.5b. How many days do you spend in the forest per week, on average, during the dry season? | Integer |
| A.5c. How many nights do you spend in the forest per week, on average, during the rainy season? | Integer |
| A.5d. How many days do you spend in the forest per week, on average, during the rainy season? | Integer |
| A.6a. How many nights do you spend in the forest away from your primary home - per week, on average, during the dry season? | Integer |
| A.6b. How many days do you spend in the forest away from your primary home - per week, on average, during the dry season? | Integer |
| A.6c. How many nights do you spend in the forest- away from your primary home - per week, on average, during the rainy season? | Integer |
| A.6d. How many days do you spend in the forest away from your primary home - per week, on average, during the rainy season? | Integer |
| A.5a. How many nights do you spend in the forest per week, on average, during the dry season? | Integer |
| A.5b. How many days do you spend in the forest per week, on average, during the dry season? | Integer |
| A.5c. How many nights do you spend in the forest per week, on average, during the rainy season? | Integer |
| A.5d. How many days do you spend in the forest per week, on average, during the rainy season? | Integer |
|  |  |
| Bite Prevention Methods |  |
| B.1a. Do you do anything to prevent mosquito bites INSIDE your house? | Yes |
|  | No |
| B.1b. If no, why not? | Text |
| B.2a. What do you do to prevent biting INSIDE your house during daytime? | Boil water |
|  | Sleep under mosquito net |
|  | Use insecticide spray |
|  | Use skin repellent |
|  | Burn coil |
|  | Burn incense or wood |
|  | Use hammock net |
|  | Use window or door screen |
|  | Use long sleeve clothing |
|  | Use Global Fund forest pack |
|  | I don’t know |
|  | Other |
| B.2b. Describe "Other" | Text |
| B.2c. What do you do to prevent biting INSIDE your house during nighttime? |  |
|  | Boil water |
|  | Sleep under mosquito net |
|  | Use insecticide spray |
|  | Use skin repellent |
|  | Burn coil |
|  | Burn incense or wood |
|  | Use hammock net |
|  | Use window or door screen |
|  | Use long sleeve clothing |
|  | Use Global Fund forest pack |
|  | I don’t know |
|  | Other |
| B.2b. Describe "Other" | Text |
| B.3a. Do you do anything to prevent mosquito bites OUTSIDE your house? | Yes |
|  | No |
| B.3b. If no, why not? | Text |
| B.4a. What do you do to prevent biting OUTSIDE your house during daytime? | Boil water |
|  | Sleep under mosquito net |
|  | Use insecticide spray |
|  | Use skin repellent |
|  | Burn coil |
|  | Burn incense or wood |
|  | Use hammock net |
|  | Use window or door screen |
|  | Use long sleeve clothing |
|  | Use Global Fund forest pack |
|  | I don’t know |
|  | Other |
| B.4b. Describe "Other" | Text |
| B.4c. What do you do to prevent biting OUTSIDE your house during nightime? | Boil water |
|  | Sleep under mosquito net |
|  | Use insecticide spray |
|  | Use skin repellent |
|  | Burn coil |
|  | Burn incense or wood |
|  | Use hammock net |
|  | Use window or door screen |
|  | Use long sleeve clothing |
|  | Use Global Fund forest pack |
|  | I don’t know |
|  | Other |
| B.4d. Describe "Other" | Text |
| B.5a. Would you like to use any other methods or products to prevent mosquito bites? | Yes |
|  | No |
| B.5b. What methods or products would you like to use to prevent mosquito bites, that you are not already using? | Text |
|  |  |
| Occupation |  |
| C1a. Which of the following activities has been a source of income for you/your family in the past year? | Farmer |
|  | Market trader |
|  | Forest collector/forager |
|  | Ranger |
|  | Driver/ motorbike taxi |
|  | Day laborer |
|  | Indigenous leader |
|  | Handicraft (basket weaving etc.) |
|  | Retired |
|  | Unemployed |
|  | Other |
| C1b. Describe "Other" | Text |
|  |  |
| Malaria Diagnosis |  |
| D1. How many times have you been diagnosed with malaria in your life? | Integer |
| D2. When was the last time you were diagnosed with malaria? | Never |
|  | Last Week |
|  | Last Month |
|  | Last 3 Months |
|  | Last 6 Months |
|  | Last Year |
|  | More than one year |
| D3a. The last time you were sick with malaria, where did you go? | Health Centre |
|  | Referral hospital |
|  | Private clinic |
|  | Pharmacy |
|  | Traditional healer |
|  | MMW or VMW |
|  | Other |
| D3b. Describe "Other" | Text |
|  |  |
| Travel |  |
| E1a. How often do you travel outside your village? | Daily |
|  | A few times a week |
|  | Weekly |
|  | Twice a month |
|  | Once a month |
|  | Less than once a month |
| E1b. What is your primary purpose for this travel? | Work in the forest |
|  | Work at the farm |
|  | Work (other location) |
|  | Visiting family/friends |
|  | Other |
| E1c. Describe other | Text |
| E2. When you travel outside your village, how far do you go on average (in hours walked)? | less than 1 hour |
|  | 1-2 hours |
|  | 2-6 hours |
|  | more than 6 hours |
| E3. What is the name of the places you most often travel to? | Text |
| E4. In the last month, have you traveled away from your home and stayed overnight? | Yes |
|  | No |
| E5. Over the past month, have you visited a health facility? | Yes |
|  | No |
